# Supplementary material for: Scavenging of reactive dicarbonyls with 2-hydroxybenzylamine reduces atherosclerosis in hypercholesterolemic Ldlr−/− mice
Source: Nat Commun. 2020 Aug 14;11:4084. doi: 10.1038/s41467-020-17915-w (PMC7429830; doi:10.1038/s41467-020-17915-w)
Supplement: Supplementary file 1 — Supplementary Information [file 41467_2020_17915_MOESM1_ESM.pdf]

Scavenging of reactive dicarbonyls with 2-hydroxybenzylamine reduces atherosclerosis in hypercholesterolemic *Ldlr*-/- mice.

Tao et al.

Supplementary Table 1: IsoLG-HOBA metabolites in tissues of *Ldlr*$^{-/-}$ mice

**Supplementary Table**		
Analytes	2-HOBA treated	4-HOBA treated
Liver (nmol/kg)		
IsoLG-HOBA-M1	0.88±0.14	ND
IsoLG-HOBA-M2	0.82±0.12	ND
IsoLG-HOBA-M3	4.17±1.85	ND
Heart (nmol/kg)		
IsoLG-HOBA-M1	0.74±0.21	ND
IsoLG-HOBA-M2	0.57±0.35	ND
IsoLG-HOBA-M3	0.24±0.17	ND
ND = not detected		

Supplementary Table 1: Levels of IsoLG-HOBA metabolites in liver and hearts of *Ldlr*$^{-/-}$ fed a Western diet for 16 weeks and continuously treated with water containing either 2-HOBA or 4-HOBA. Structures for metabolites 1-3 (M1, M2, M3) are shown in Supplementary Figure 6. No signal for IsoLG-HOBA metabolites were detected in mice treated with 4-HOBA. n = 3 biologically independent mice per group. Data are presented as mean ± SEM. Source data are provided as a source data file.

Supplementary Figure 1

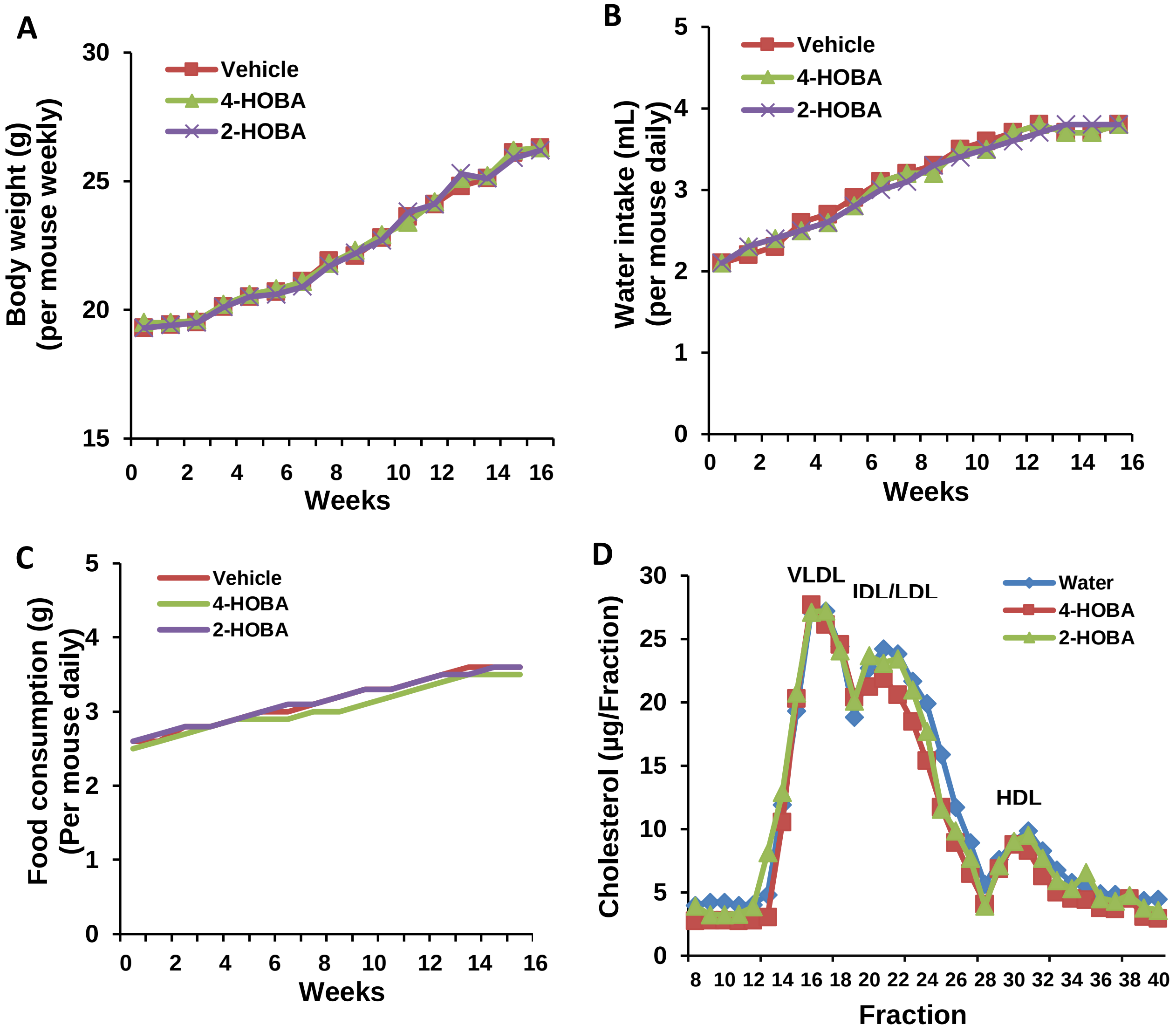

Supplementary Figure 1. 2-HOBA does not impact body weight, water intake, food consumption or lipoprotein profile in hypercholesterolemic *Ldlr*-/- mice. (A-D) *Ldlr*-/- mice were fed a Western diet for 16 weeks and treated with 1 g/L 2-HOBA, 4-HOBA, or vehicle.(A-C) Body weight (A), water intake (B), and diet consumption (C) were measured. n = 10 biologically independent mice per group. Data are presented as the mean. B, Water intake was measured in the *Ldlr*-/- mice. Biologically independent mice used n=10 in vehicle, n=10 in 2-HOBA group, n=10 in 4-HOBA group. The data are presented as mean. D) The plasma from hypercholesterolemic *Ldlr*-/- mice that were fasted for 6 hours was pooled, and fast performance liquid chromatography (FPLC) was performed using a Superose 6 column. Total cholesterol was measured using an enzymatic assay. Four mouse plasma samples were pooled per group of mice. n = 2 two biologically independent experiments. The data are presented as the mean. Source data are provided as a source data file.

Supplementary Figure 2

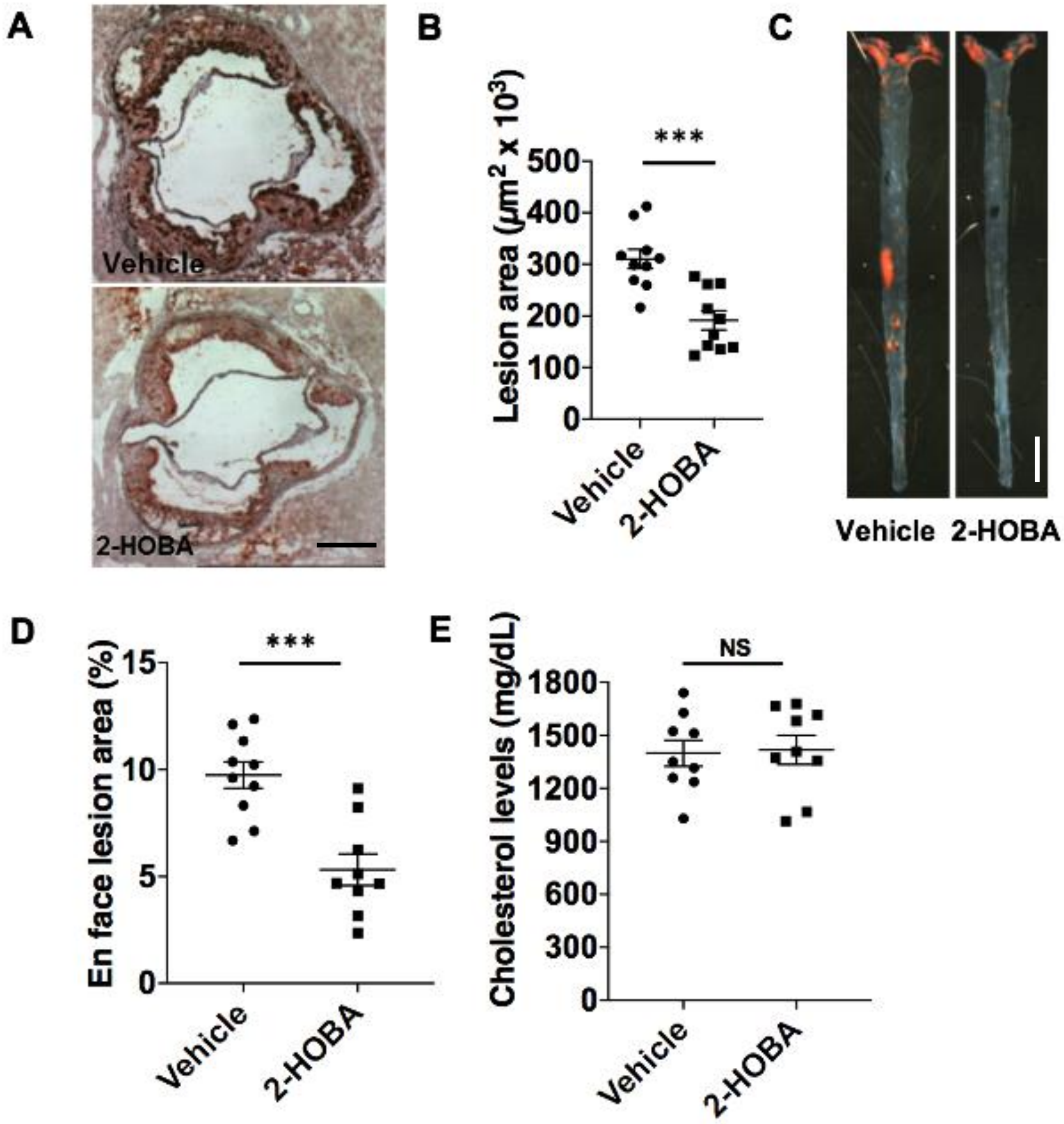

Supplementary Figure 2. 2-HOBA reduces atherosclerotic lesions in hypercholesterolemic male *Ldlr*-/- mice. 12-week old male *Ldlr*-/- mice were pretreated with 1 g/L 2-HOBA or vehicle (water) for 2 weeks and then the treatment was continued for 16 weeks during which the mice were fed a Western diet. (A-B) Representative images (A) and quantitation (B) of Oil-Red-O staining in proximal aorta root sections. Scale bar = 500 µm. n = 10 biologically independent mice per group. Data are presented as mean ± SEM. Two-sided unpaired *t* test, p-value for 2-HOBA vs vehicle is ***0.0003. (C-D) Representative images (C) and quantitation (D) of Oil-Red-O staining in open-pinned aortas. Scale bar = 500 µm. n = 10 (vehicle) or 9 (2-HOBA) biologically independent mice. Data are presented as mean ± SEM. Two-sided unpaired *t* test, p-value for 2-HOBA vs vehicle is ***0.0002. (E) 2-HOBA does not affect the cholesterol levels of male *Ldlr*-/- mice. n = 9 biologically independent mice per group. Data are presented as mean ± SEM. Two-sided unpaired *t* test, p-value for 2-HOBA vs vehicle is 0.6665, NS, not significant. Source data are provided as a source data file.

Supplementary Figure 3

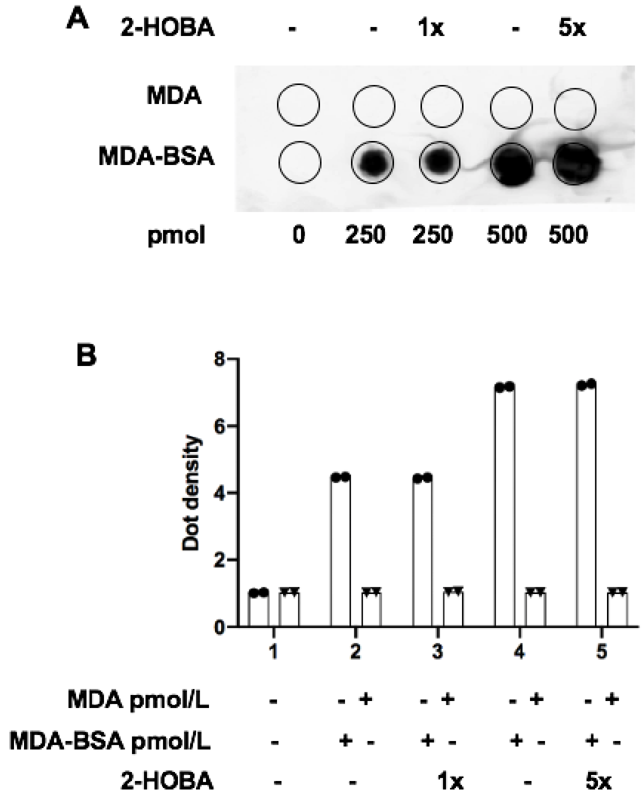

Supplementary Figure 3. 2-HOBA does not impact anti-MDA antibody interaction with MDA-BSA. A series of doses of MDA-BSA or MDA alone were incubated with 1x or 5x 2-HOBA. Then 2 µl of each sample was loaded onto HyBond-C membrane, and incubated with the blocking buffer, primary anti-MDA antibody and fluorescent seconary antibody after vigorous washing. The image was captured by the Odyssey system (A) and quantitated by ImageJ software (B). n = 2 biologically independent experiments per group. The data are presented as the mean. Source data are provided as a source data file.

Supplementary Figure 4

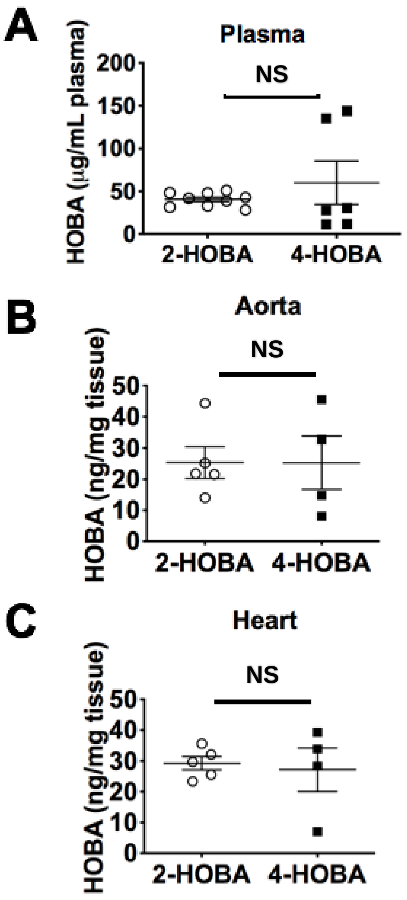

Supplementary Figure 4. The concentration of 2-HOBA or 4-HOBA was measured in plasma and tissues from *Ldlr*-/- mice. A) Eight week old male *Ldlr*-/- mice were fed WD for 16 weeks and were continuously treated with water containing either 2-HOBA or 4-HOBA. Plasma was collected 30 min after oral gavage of mice with either 2-HOBA or 4-HOBA (5 mg each mouse). n = 9 (2-HOBA) or 6 (4-HOBA) biologically independent mice. Data are presented as mean ± SEM. Two-sided Mann-Whitney Test, p-value for 2-HOBA vs. 4-HOBA is 0.3884, NS, not significant. (B-C) Levels of HOBA were measured in the aorta and heart of male *Ldlr*-/- mice consuming a chow diet 30 min after oral gavage of 2-HOBA or 4-HOBA (5 mg each mouse). n = 5 (2-HOBA) or 4 (4-HOBA) biologically independent mice. Data are presented as mean ± SEM. Two-sided Mann-Whitney Test, p-values for 2-HOBA vs. 4-HOBA are >0.9999 (B) and 0.9048 (C), NS, not significant. Source data are provided as a source data file.

Supplementary Figure 5

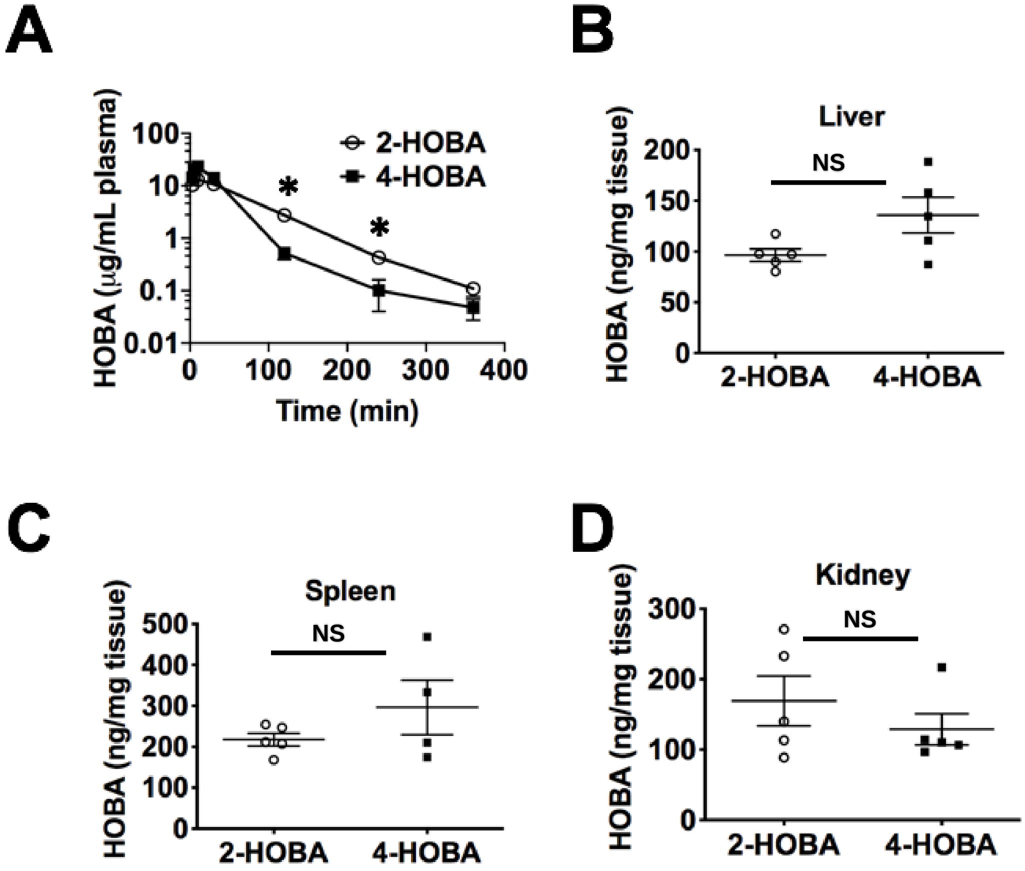

Supplementary Figure 5. The levels of 2-HOBA or 4-HOBA in plasma and tissues from C57BL/6J mice. (A) Plasma samples were collected from female C57BL/6J mice on a chow diet after intraperitoneal injection of 1 mg of 2-HOBA or 4-HOBA. The levels of 2-HOBA or 4-HOBA were measured using LC/MS as described in the Methods. n = 3 biologically independent mice per group. Data are presented as mean ± SEM. Two-sided paired *t* test, p-values for 2-HOBA vs. 4-HOBA are 0.114, 0.0977, 0.1519, 0.3204, *0.0206, *0.0308, and 0.0814 at 3min, 5min, 10min, 30min, 120min, 240min, and 360min, respectively, by using paired *t* test, respectively. * indicates p<0.05. (B-D) The levels of 2-HOBA and 4-HOBA in the liver (B), spleen (C), and kidney (D) were measured 30min after female C57BL/6J mice on a chow diet were intraperitoneally injected with 1 mg. (B and D) n = 5 biologically independent mice per group. (C) n = 5 (2-HOBA) or 4 (4-HOBA) biologically independent mice. (B-D) Data are presented as mean ± SEM. Two-sided Mann-Whitney Test, p-values for 2-HOBA vs. 4-HOBA are 0.1508 (B), 0.5556 (C), and 0.4206 (D), NS,not significant. Source data are provided as a source data file.

Supplementary Figure 6

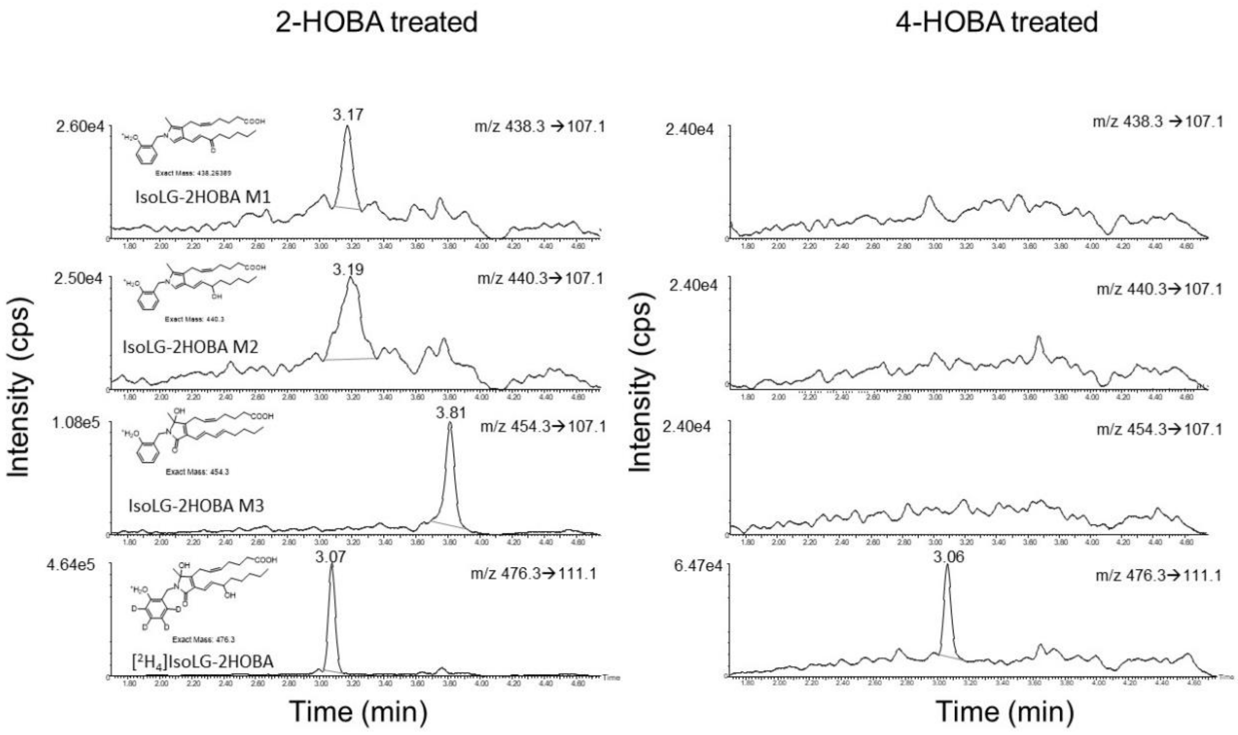

Supplementary Figure 6. Detection of metabolites of isolevuglandin modified 2-HOBA (IsoLG-2-HOBA) in liver of 2-HOBA treated *Ldlr*-/- mice. Putative metabolites were identified as described in Methods. Representative chromatographs for livers from mice treated with 2-HOBA (left) and 4-HOBA (right) are shown for the three most abundant IsoLG-HOBA metabolites (three upper panels) and the internal standard (lower panel). One potential structure of each metabolite is shown on the left of the chromatograph.

Supplementary Figure 7

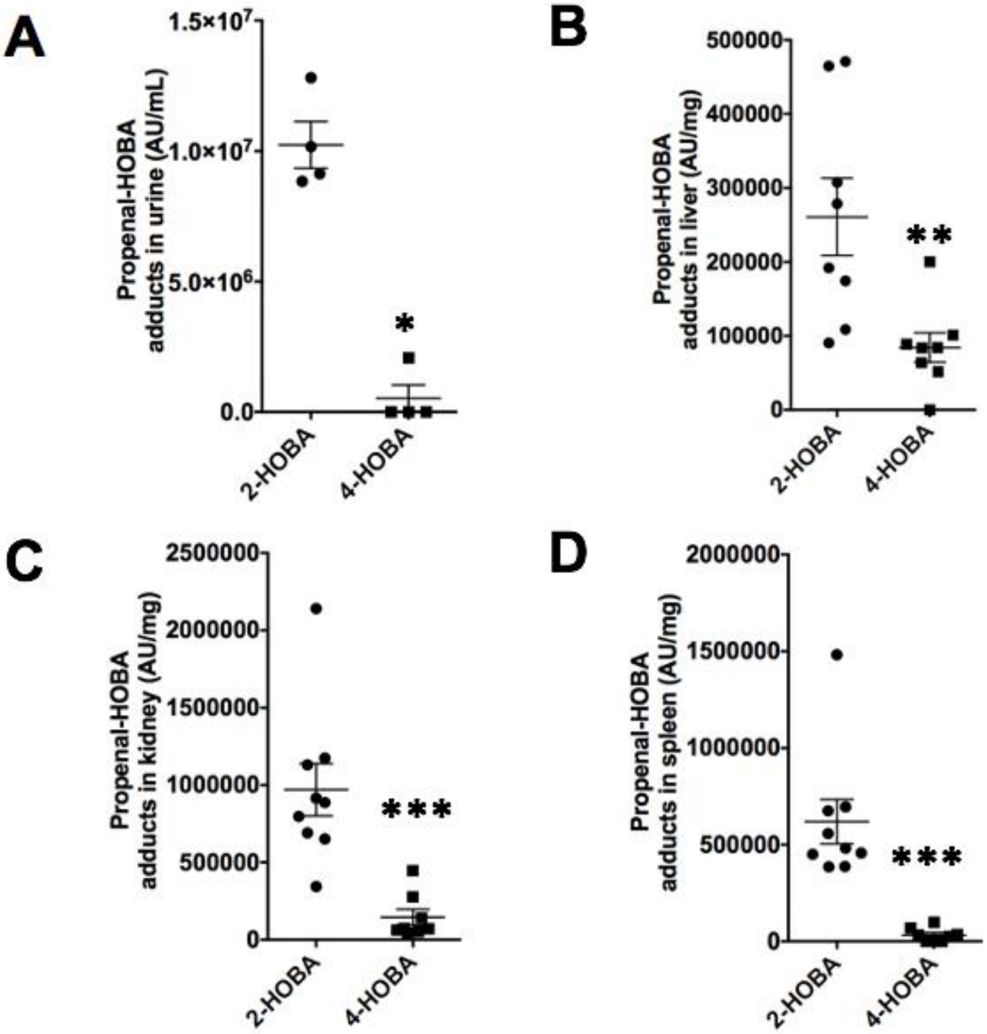

Supplementary Figure 7. MDA-2-HOBA adducts versus MDA-4-HOBA adducts were more readily formed in vivo. (A-D) Urine samples (A) were collected for 16 h after oral gavage of male *Ldlr-/-* mice on a WD with either 5 mg of 2-HOBA or 4-HOBA. After 16 h , the *Ldlr-/-* mice were sacrificed and tissues harvested (B-D). Urine and tissue HOBA-propenal adducts were measured using LC-MS/MS as described in Methods. (A) The HOBA-propenal adducts in urine. n = 4 biologically independent urine samples per group. Data are presented as mean ± SEM. Two-sided Mann-Whitney Test, p-value for 2-HOBA vs. 4-HOBA is *0.0286. (B) The HOBA-propenal adducts in liver. n = 8 biologically independent mice per group. Data are presented as mean ± SEM. Two-sided Mann-Whitney Test, p-value for 2-HOBA vs. 4-HOBA is **0.003. (C) The HOBA-propenal adducts in kidney. n = 9 (2-HOBA) or 8 (4-HOBA) biologically independent mice. Data are presented as mean ± SEM. Two-sided Mann-Whitney Test, p-value for 2-HOBA vs. 4-HOBA is ***0.0002. (D) The HOBA-propenal adducts in spleen. n = 9 (2-HOBA) or 8 (4-HOBA) biologically independent mice. Data are presented as mean ± SEM. Two-sided Mann-Whitney Test, p-value for 2-HOBA vs. 4-HOBA is ***0.0001. Source data are provided as a source data file.

Supplementary Figure 8

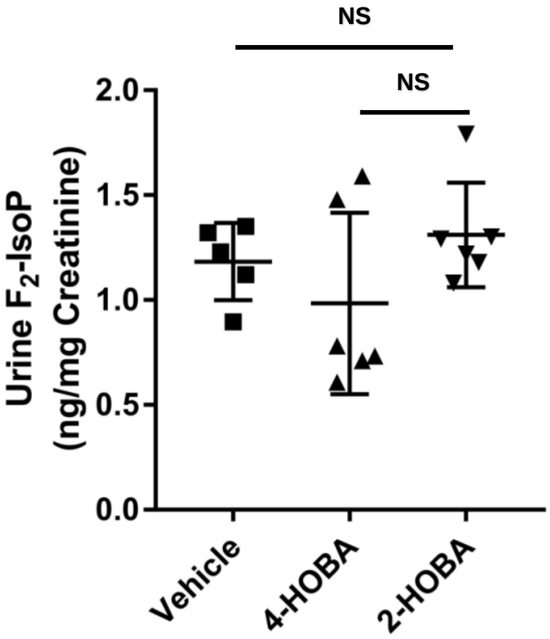

Supplementary Figure 8. 2-HOBA does not impact urine F2-IsoP in hypercholesterolemic *Ldlr*-/- mice. The urine F2-IsoP levels were measured by LC/MS/MS from *Ldlr*-/- mice consuming a western diet for 16 weeks and treated with 1 g/L 2-HOBA, 4-HOBA, or vehicle alone. Urinary creatinine levels were measured for normalization. n = 5 (vehicle) or 6 (4-HOBA and 2-HOBA) biologically independent urine samples. Data are presented as mean ± SD. Kuriskal-Wallis, One-way ANOVA on ranks test, p-value is 0.43, NS, not significant. Source data are provided as a source data file.

Supplementary Figure 9

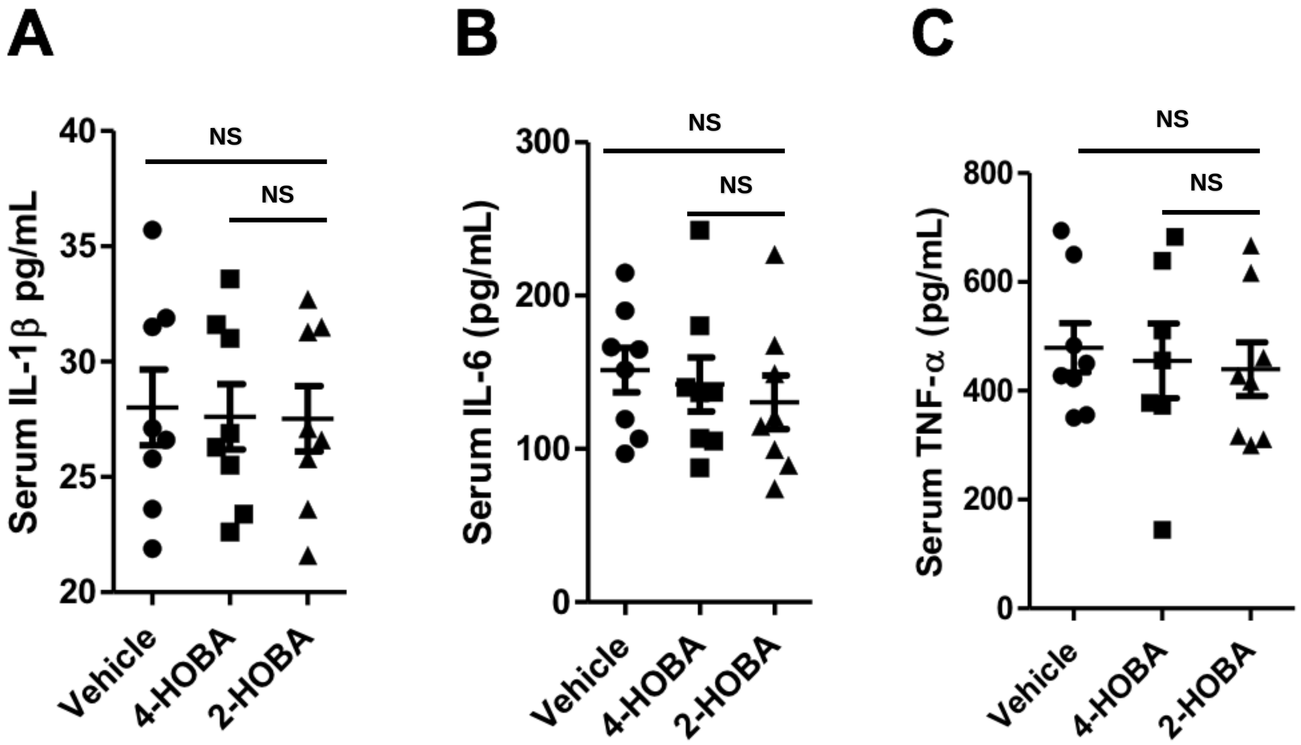

Supplementary Figure 9. Levels of cytokines in serum of *Ldlr*-/- mice fed a chow diet for 6 weeks and continuously treated with water alone or containing 1g/L of either 2-HOBA or 4-HOBA. (A-C) Serum IL-1β, IL-6 and TNF-α levels were measured by ELISA (R&D System). (A-C) n = 8 biologically independent mice per group. Data are presented as mean ± SEM. One-way ANOVA with Bonferroni's post hoc test, p-values for all comparisons are >0.999, NS, not significant. Source data are provided as a source data file.

Supplementary Figure 10

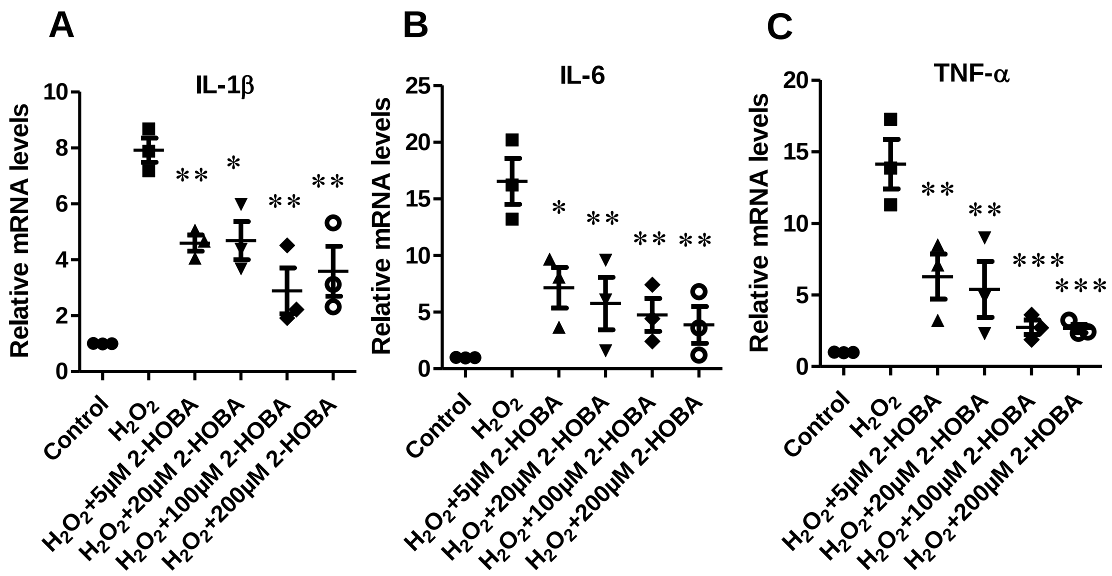

Supplementary Figure 10. 2-HOBA reduces expression of inflammatory cytokines by H_2O_2-stimulated macrophages. WT macrophages were treated with or without 100 µM H_2O_2 with or without increasing concentrations of 2-HOBA for 24 hours. Total RNA was isolated and purified, cDNA was synthesized, and the mRNA levels of IL-1β (A), IL-6 (B) and TNF-α (C) were measured by real-time PCR. (A-C) n = 3 biologically independent cell culture experiments. Data are presented as mean ± SEM. One-way ANOVA with Bonferroni's post hoc test. (A) p-values of H_2O_2 vs. 5 µm 2-HOBA, 20 µm 2-HOBA, 100 µm 2-HOBA, and 200 µm 2-HOBA are **0.0113, *0.0135,** 0.0004, and **0.0015. (B) p-values of H_2O_2 vs. 5 µm 2-HOBA, 20 µm 2-HOBA, 100 µm 2-HOBA, and 200 µm 2-HOBA are *0.0107, **0.0038, **0.0019, and **0.0010. (C) p-values of H_2O_2 vs. 5 µm 2-HOBA, 20 µm 2-HOBA, 100 µm 2-HOBA, and 200 µm 2-HOBA are **0.0043, **0.0018, ***0.0002 and ***0.0002. Source data are provided as a source data file.

Supplementary Figure 11

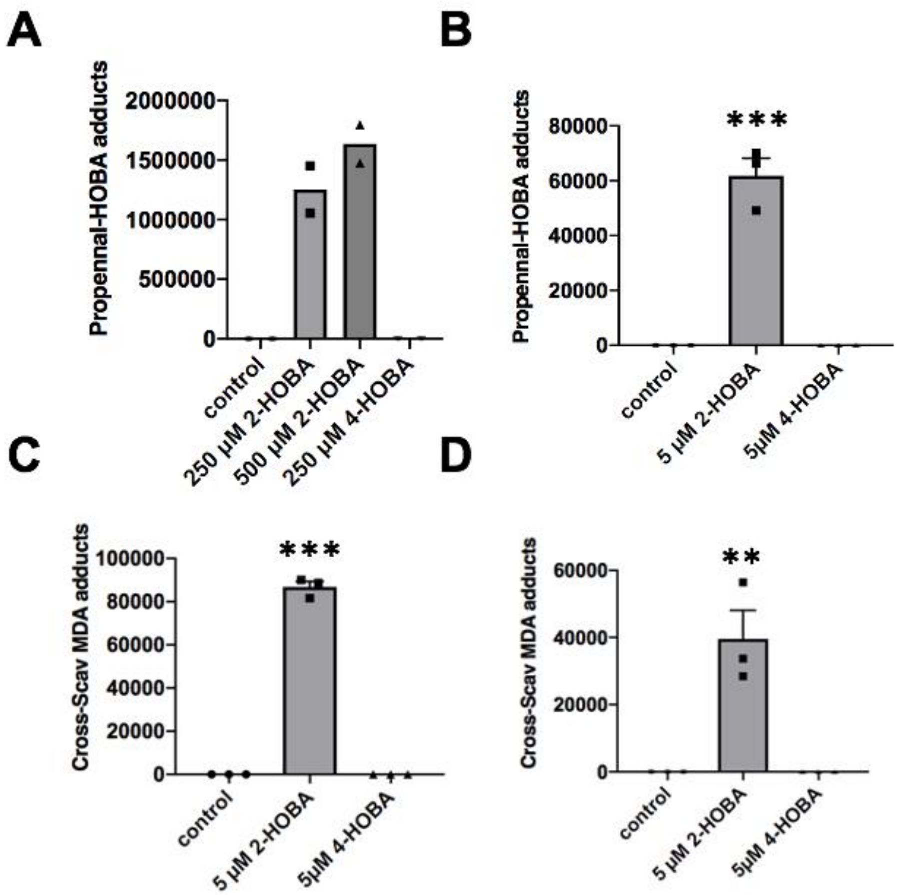

Supplementary Figure 11. Treatment of macrophages with 2-HOBA results in formation of 2-HOBA-MDA adducts. Peritoneal macrophages were isolated from C57BL/6J mice and incubated with 50 µg/mL ox-LDL, and treated with either 250 µm 2-HOBA or 4-HOBA (A) or 5µm 2-HOBA or 4-HOBA (B, C, D) for 24h. Cell samples were collected and the HOBA-MDA adducts were measured using LC-MS/MS as described in Methods. (A) n = 2 biologically independent experiments. Data are presented as the mean. (B-D) n = 3 biologically independent experiments. Data are presented as mean ± SEM. One-way ANOVA with Bonferroni's post hoc test. (B) p-values of control vs. 5 µM 2-HOBA and 5 µM 4-HOBA are ***<0.0001 and >0.9999. (C) p-values of control vs. 5 µM 2-HOBA and 5 µM 4-HOBA are ***<0.0001 and >0.9999. (D) p-values of controls vs. 5 µM 2-HOBA and 5 µM 4-HOBA are **0.004 and >0.9999. Source data are provided as a source data file. C & D. Vertical axis label: Cross-Scav (MDA-crosslinked HOBA scavenger).

Supplementary Figure 12

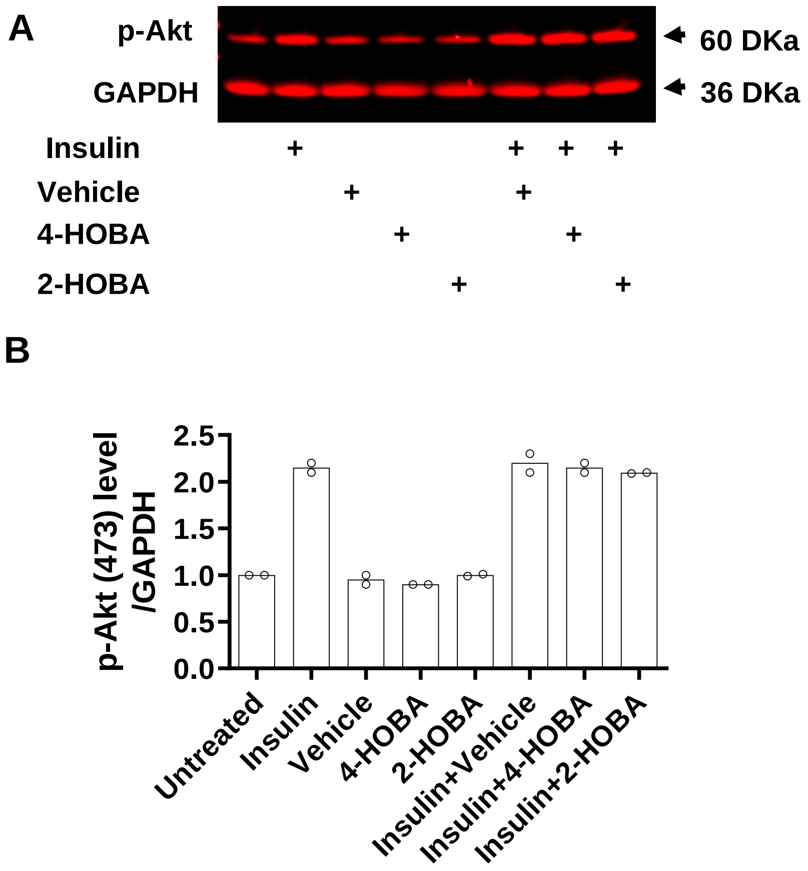

Supplementary Figure 12. 2-HOBA does not influence Akt signaling in macrophages. (A-B) WT macrophages were treated with or without vehicle (water), 250 µM 4-HOBA or 2-HOBA for 1 hour, and then incubated with or without 100 nM insulin as indicated for 15 min. Phospho-Akt (S473) and GAPDH were detected by Western Blotting. The band density was quantitated by ImageJ software (B). n = 2 biologically independent experiments. The data are presented as the mean. Source data are provided as a source data file.

Supplementary Figure 13

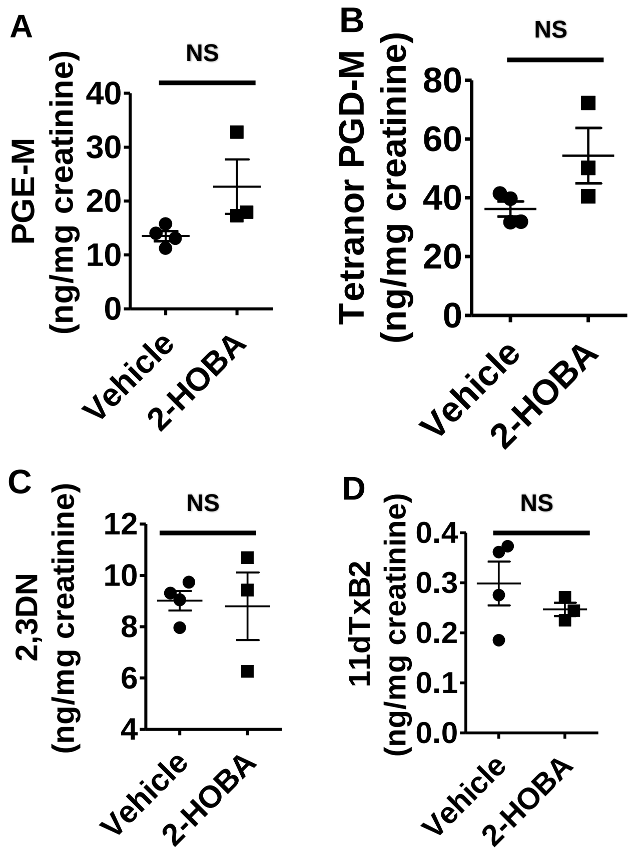

Supplementary Figure 13. Effect of 2-HOBA on prostaglandin metabolites. (A-D) Male *Ldlr*-/- mice were pretreated with 1 g/L 2-HOBA or vehicle (water) for 2 weeks and then the treatment was continued during 12 weeks of consuming a western diet. Urine samples were then collected in metabolic cages with 2 mice per cage and the prostaglandin metabolites analyzed by LC/MS/MS as described in Methods. Creatine was measured for normalization. (A-D) n = 4 (vehicle) or 3 (2-HOBA) biologically independent urine samples. Data are presented as mean ± SEM. Two-sided Mann-Whitney Test, p-values for vehicle vs. 2-HOBA are 0.0571 (A), 0.1143 (B), 0.8571 (C), and 0.40 (D), NS, not significant. Source data are provided as a source data file.

Supplementary Figure 14

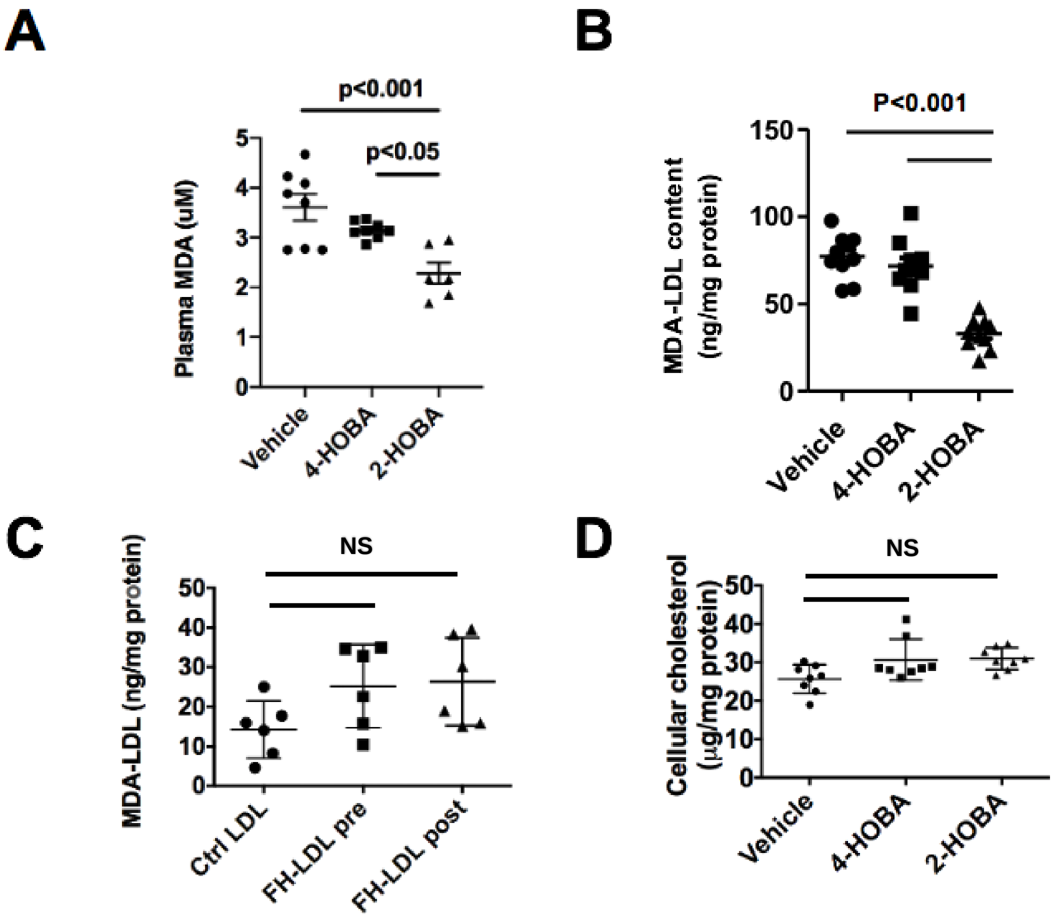

Supplementary Figure 14. Effects of 2-HOBA on plasma and LDL MDA adducts in hypercholesterolemic *Ldlr-/-* mice. (A, B, and D) *Ldlr-/-* mice were fed a Western diet for 16 weeks and treated with 2-HOBA, 4-HOBA, or vehicle. (A) The MDA content in plasma was measured by TBARS Assay. n = 8 (vehicle and 4-HOBA) or 6 (2-HOBA) biologically independent mice. Data are presented as mean ± SEM. One-way ANOVA with Bonferroni's post hoc test, p-values of 2-HOBA vs 4-HOBA and 2-HOBA vs vehicle are 0.024 and 0.0007. (B) The levels of MDA adducts were measured in LDL isolated from the *Ldlr-/-* mice by ELISA. n = 10 biologically independent mice per group. Data are presented as mean ± SEM. One-way ANOVA with Bonferroni's post hoc test, p-values of 2-HOBA vs 4-HOBA and 2-HOBA vs vehicle are <0.0001. (C) For comparison, LDL was isolated from control and FH subjects pre and post LDL apheresis and the MDA content measured by ELISA. n = 6 biologically independent humans per group. Data are presented as mean ± SEM. One-way ANOVA with Bonferroni's post hoc test, p-values of control LDL vs FH-LDL pre LDL apheresis, control LDL vs FH-LDL post LDL aphersis, and FH-LDL pre LDL aphersis vs FH-LDL post LDL aphersis are 0.2176, 0.1483, and >0.9999, NS, not significant. (D) LDL was isolated from 2-HOBA, 4-HOBA, or vehicle treated hypercholesterolemic *Ldlr-/-* mice. WT peritoneal macrophages were incubated for 24 hrs with the LDL and the cellular cholesterol content was measured as described in Methods. n = 8 biologically independent mice per group. Data are presented as mean ± SEM. One-way ANOVA with Bonferroni's post hoc test, p-values of 2-HOBA vs 4-HOBA and 2-HOBA vs vehicle are >0.9999 and 0.0516, NS, not significant. Source data are provided as a source data file.

Supplementary Figure 15

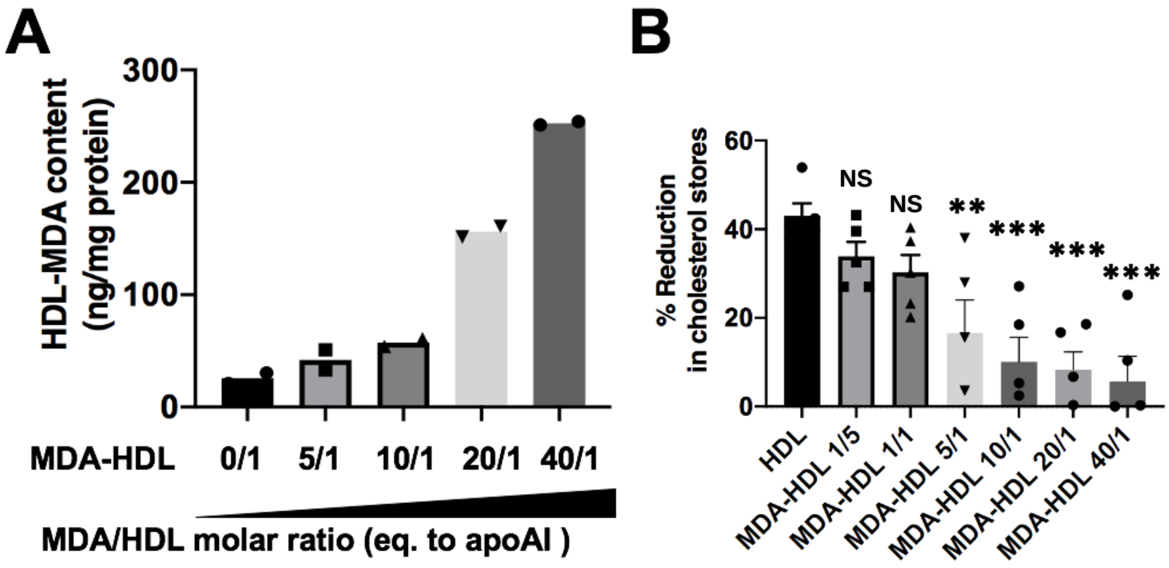

Supplementary Figure 15. Modification of HDL with increasing concentrations of MDA impaired cholesterol efflux in a dose dependent manner. (A) The HDL was modified with MDA and the MDA adducts were measured by ELISA. n = 2 biologically independent experiments. Data are presented as the mean. (B) *Apoe*$^{-/-}$ peritoneal macrophages were incubated with ac-LDL for 40h and then incubated for 24h with 50ug/mL of control HDL or MDA-HDL. The net cholesterol efflux capacity was measured as described in Methods. n = 2 biologically independent experiments. Data are presented as mean ± SEM. One-way ANOVA with Bonferroni's post hoc test, p-values of control HDL vs. MDA-HDL 1/5, MDA-HDL 1/1, MDA-HDL 5/1, MDA-HDL 10/1, MDA-HDL 20/1, and MDA-HDL 40/1 are >0.05, >0.05, **0.0041, ***0.0003, ***0.0002, and ***0.0001, respectively. NS, not significant. Source data are provided as a source data file.

Supplementary Figure 16

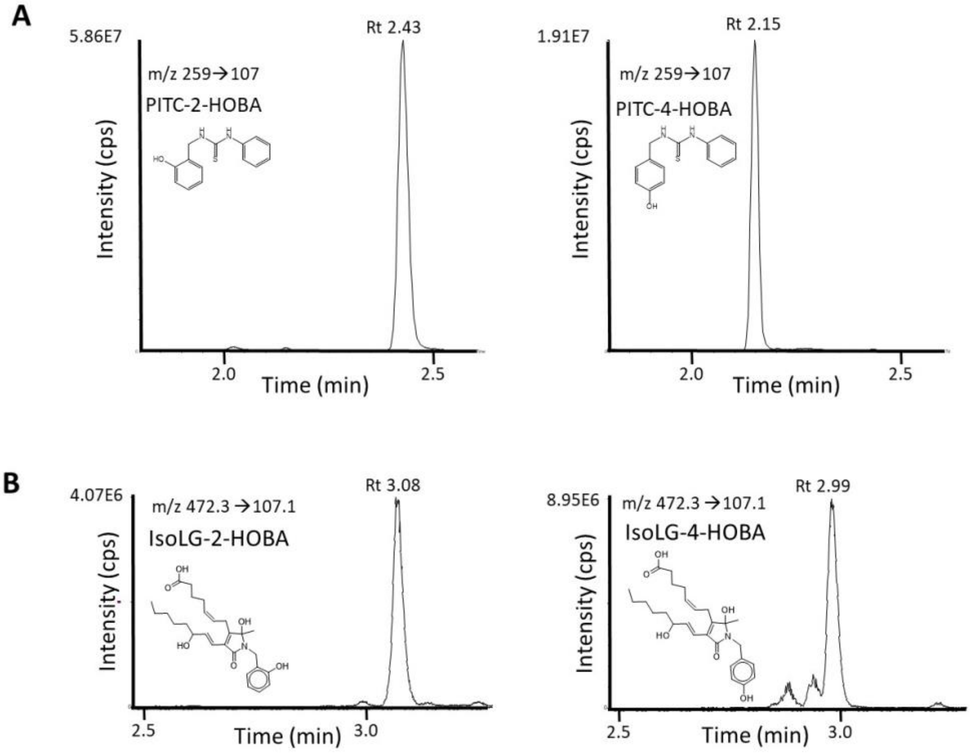

Supplementary Figure 16. The same multiple reaction monitoring (MRM) parameters that detect 2-HOBA aldehyde adducts also detect 4-HOBA aldehyde adducts. Panel A: MRM *m/z 259* → *m/z 107* chromatograph for PITC derivatized 2-HOBA (left) and PITC derivatized 4-HOBA (right). Panel B: MRM chromatograph *m/z 472* → *m/z 107* for IsoLG(hydroxylactam)-2-HOBA (left) and IsoLG(hydroxylactam)-4-HOBA (right). The MRM chromatographs for MDA(propenal)-2-HOBA adduct and MDA(propenal)-4-HOBA adduct have been previously published.

Supplementary Figure 17

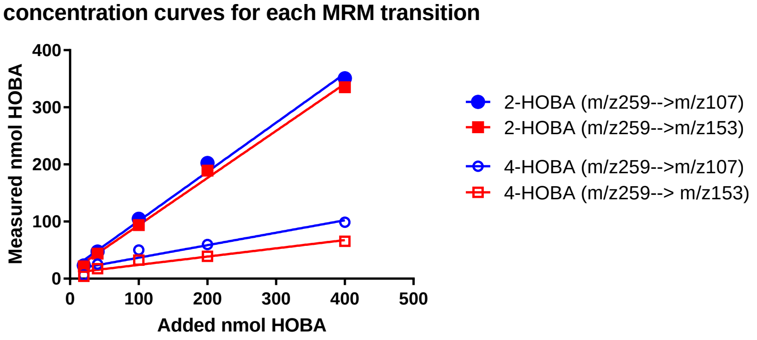

	4-HOBA (m/z259--> m/z153)	4-HOBA (m/z259-->m/z107)	2-HOBA (m/z259-->m/z153)	2-HOBA (m/z259-->m/z107)
Best-fit values ± SE				
Slope	0.1447 ± 0.02288	0.2183 ± 0.03522	0.8247 ± 0.02901	0.8597 ± 0.03757
Y-intercept	9.712 ± 4.712	14.95 ± 7.251	11.29 ± 5.973	15.1 ± 7.737
X-intercept	-67.13	-68.47	-13.69	-17.56
1/slope	6.912	4.58	1.213	1.163

correction factor for 4-HOBAwhen using m/z 153 transistion = 6.912/1.213 = 5.7
correction factor for 4-HOBA when using m/z 107 transistion 4.580/1.163 = 3.9

Supplementary Figure 17. The concentration response curve for the PITC derivative of 4-HOBA differs from that of 2-HOBA when [$_2$H$_4$]2-HOBA is used as an internal standard and therefore requires use of a correction factor. Varying concentrations (20-400 nmol) of either 2-HOBA or 4-HOBA were mixed with 1 nmol of [$_2$H$_4$]2-HOBA, the compounds derivatized with PITC, and then analyzed on LC/MS using either MRM transition m/z 259→m/z 107 or m/z 259 → 153 for 2-HOBA and 4-HOBA and either m/z 263 → m/z 111 or m/z 263 → 153 for [$_2$H$_4$]2-HOBA and the measured nmol calculated using the ratio of peak areas. The concentration response slope for each was calculated using GraphPad Prism, and the correction factor for 4-HOBA calculated as the ratio of the two slopes. n = 3 biologically independent experiments n=3 per group. Data are presented as the mean. Source data are provided as a source data file.

Supplementary Figure 18

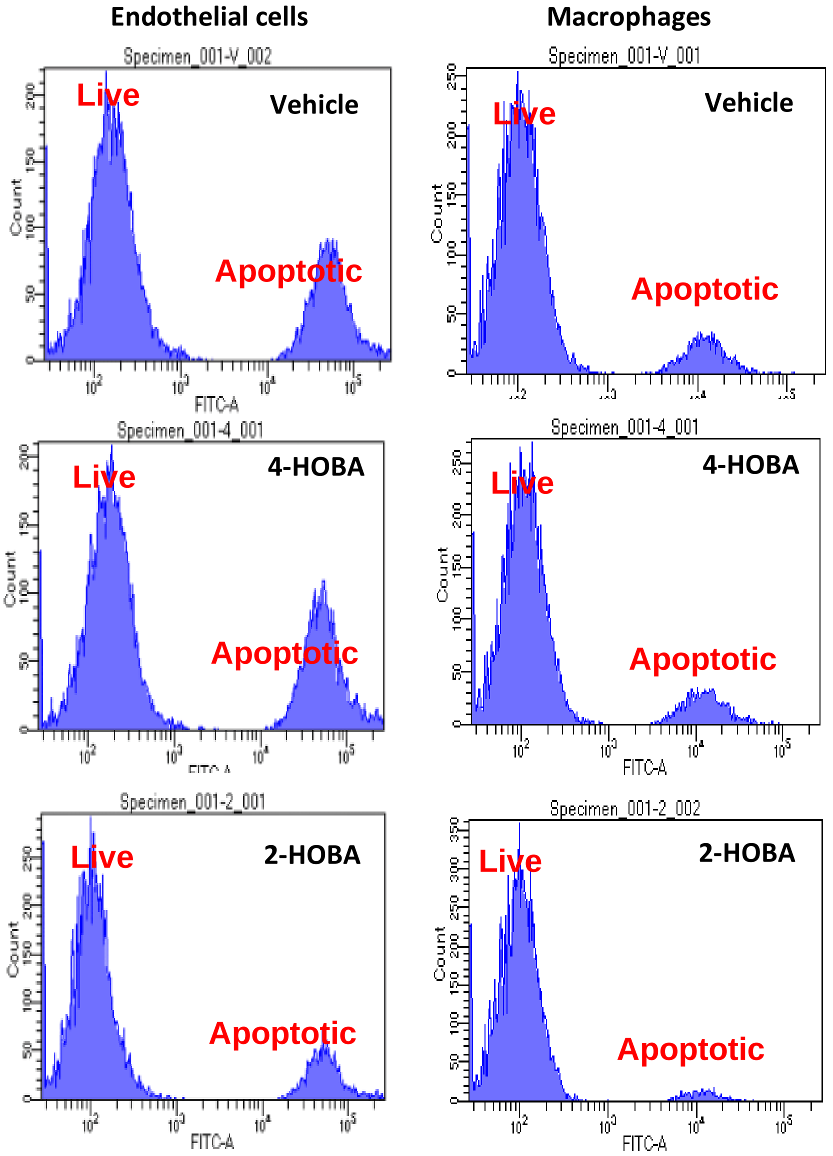

Supplementary Figure 18. Representative pictures of gating strategy to detect Anexin V positive apoptotic cells in Figure 6A and 6B. The cells were treated with Alera Fluo 488 Anexin V for 15 min followed by flow cytotemtry analysis. A 3-laser BD LSRII (BD Biosciences) configured with 405 nm, 488 nm, and 633 nm lasers was used. The BD LSRFortessaTM was the main analytical platform used for flow cytometry, and BD Biosciences digital flow cytometry software was used. For each experiment, positive and negative controls were prepared and applied for validation. Based on their forward and side scatter properties, the Annexin V Alexa Fluo 488 positive (Apoptotic cells) or negative cells (Live cells) were determined in gating.
