# Supplementary material for: Scavenging of reactive dicarbonyls with 2-hydroxybenzylamine reduces atherosclerosis in hypercholesterolemic Ldlr−/− mice
Source: Nat Commun. 2020 Aug 14;11:4084. doi: 10.1038/s41467-020-17915-w (PMC7429830; doi:10.1038/s41467-020-17915-w)
Supplement: Supplementary file 3 — Reporting Summary [file 41467_2020_17915_MOESM3_ESM.pdf]

nature research

Corresponding author(s): MacRae F. Linton

Last updated by author(s): Jun 26, 2020

Reporting Summary

Nature Research wishes to improve the reproducibility of the work that we publish. This form provides structure for consistency and transparency in reporting. For further information on Nature Research policies, see Authors & Referees and the Editorial Policy Checklist .

Statistics

For all statistical analyses, confirm that the following items are present in the figure legend, table legend, main text, or Methods section.

n/a	Confirmed	
☐	☒	The exact sample size (*n*) for each experimental group/condition, given as a discrete number and unit of measurement
☐	☒	A statement on whether measurements were taken from distinct samples or whether the same sample was measured repeatedly
☐	☒	The statistical test(s) used AND whether they are one- or two-sided<br>*Only common tests should be described solely by name; describe more complex techniques in the Methods section.*
☐	☒	A description of all covariates tested
☐	☒	A description of any assumptions or corrections, such as tests of normality and adjustment for multiple comparisons
☐	☒	A full description of the statistical parameters including central tendency (e.g. means) or other basic estimates (e.g. regression coefficient) AND variation (e.g. standard deviation) or associated estimates of uncertainty (e.g. confidence intervals)
☐	☒	For null hypothesis testing, the test statistic (e.g. *F*, *t*, *r*) with confidence intervals, effect sizes, degrees of freedom and *P* value noted<br>*Give P values as exact values whenever suitable.*
☒	☐	For Bayesian analysis, information on the choice of priors and Markov chain Monte Carlo settings
☒	☐	For hierarchical and complex designs, identification of the appropriate level for tests and full reporting of outcomes
☒	☐	Estimates of effect sizes (e.g. Cohen's *d*, Pearson's *r*), indicating how they were calculated

Our web collection on statistics for biologists contains articles on many of the points above.

Software and code

Policy information about availability of computer code

Data collection	The KS300 imaging system (Kontron Elektronik GmbH) and Slidebook6 (Intelligent Imaging Innovations, Inc.) were used in acquiring image data for atherosclerosis, Odyssey 3.0 Quantification software (LI-COR) was used to obtain the image from Western Blot.
Data analysis	GraphPad Prism version 5 and 7 and KS300 imaging system (Kontron Elektronik GmbH), and NIH ImageJ bundled with 64-bit Java 1.8.0_112 under open source license (https://Data analysis imagej.nih.gov/ij/) were applied for data analysis.

For manuscripts utilizing custom algorithms or software that are central to the research but not yet described in published literature, software must be made available to editors/reviewers. We strongly encourage code deposition in a community repository (e.g. GitHub). See the Nature Research guidelines for submitting code & software for further information.

Data

Policy information about availability of data

All manuscripts must include a data availability statement. This statement should provide the following information, where applicable:

- Accession codes, unique identifiers, or web links for publicly available datasets
- A list of figures that have associated raw data
- A description of any restrictions on data availability

All data included in this publication has been provided in the Source data file. Additional data that support the findings of this study are available from the corresponding author upon reasonable request.

Field-specific reporting

Please select the one below that is the best fit for your research. If you are not sure, read the appropriate sections before making your selection.

Life sciences study design

All studies must disclose on these points even when the disclosure is negative.

Sample size	Sample sizes of the atherosclerosis studies were based on power calculations. Sample size estimates were gated on the number of animals needed to see a 30% decrease of atherosclerotic lesion area with a standard deviation of 10% of the mean, in both atherosclerotic prevention and immunohistochemical studies. Using calculations with alpha 0.05 and power 80%, the required sample size was calculated to be 6 mice per group. Sample sizes of >6 were used for most experiments. The sample size of the human subjects was based on availability of subjects with severe familial hypercholesterolemia undergoing LDL apheresis.
Data exclusions	No exclusions
Replication	Independent experiments were performed as presented in methods, and the in vitro studies were performed in triplicate as indicated in the manuscript. Sample size was also described in the manuscript to ensure reproducibility.
Randomization	Mice were matched across treatment groups based on body weight, birth date then randomly assigned to different control or treatment groups. Sex and age matched mice were randomly assigned into control and treatment groups. Male and female mice were studied in separate groups in the atherosclerosis studies.
Blinding	Blinding was not possible for most in vitro experiments as group allocation and treatment was administered by the researcher collecting the data. However, for histological examination samples were de-identified and therefore blinded to the technician and pathologist performing the examination.

Reporting for specific materials, systems and methods

We require information from authors about some types of materials, experimental systems and methods used in many studies. Here, indicate whether each material, system or method listed is relevant to your study. If you are not sure if a list item applies to your research, read the appropriate section before selecting a response.

Materials & experimental systems

n/a	Involved in the study	
☐	☒	Antibodies
☐	☒	Eukaryotic cell lines
☒	☐	Palaeontology
☐	☒	Animals and other organisms
☐	☒	Human research participants
☒	☐	Clinical data

Methods

n/a	Involved in the study	
☒	☐	ChIP-seq
☐	☒	Flow cytometry
☒	☐	MRI-based neuroimaging

Antibodies

Antibodies used	Mouse plasma was immunoprecipitated with 10 µg of polyclonal antibody goat anti-mouse ApoAI (Novus Biologicals, Cat#: NB600-609). Membranes were probed with primary antibodies rabbit anti- mouse ApoAI (Novus Biologicals, Cat#: NBP2-52979, dilution fold:1/1000) or Rabbit anti-mouse MDA (Abcam cat# ab646, dilution fold:1/1000), Rabbit anti Phospho-Akt (Ser473) Antibody #9271 (Cell signaling, dilution fold:1/1000), mouse GAPDH Antibody (1D4)(Novus, Cat#NB300_211, dilution fold 1/2000), CD68 (Abcam, Cat#134351, dilution fold 1/100) and fluorescent tagged IRDye 680 (LI-COR) secondary antibody as described in the methods.
Validation	The antibodies were validated by the manufactures as shown in the websites.<br>https://www.novusbio.com/products/apolipoprotein-a-i-apoa1-antibody_nb600-609.<br>https://www.novusbio.com/products/apolipoprotein-a-i-apoa1-antibody_nbp2-52979<br>https://www.abcam.com/malondialdehyde-antibody-ab6463.html<br>https://www.cellsignal.com/products/primary-antibodies/phospho-akt-ser473-antibody/9271<br>https://www.novusbio.com/products/gapdh-antibody-1d4_nb300-221<br>https://www.abcam.com/cd68-antibody-y182a-fitc-ab134351.html

Eukaryotic cell lines

Policy information about cell lines

Cell line source(s)	Human aortic endothelial cells (HAECs) were obtained from Lonza. We used peritoneal macrophages from mice rather than mouse J774 macrophage cell line.

Authentication	HAECs were obtained from Lonza and maintained in endothelial cell basal medium-2 plus 1% FBS and essential growth factors (Lonza).
Mycoplasma contamination	All cell lines tested negative for mycoplasma contamination
Commonly misidentified lines (See ICLAC register)	No commonly misidentified cell lines were used in the study.

Animals and other organisms

Policy information about studies involving animals; ARRIVE guidelines recommended for reporting animal research

Laboratory animals	Ldlr-/- and WT on C57BL/6 background mice were obtained from the Jackson Laboratory. Mice were group housed (3-4/cage) in a humidity and temperature-controlled room with 12h dark/light cycle in a facility with automated temperature, humidity, and light cycle control. Animal protocols were performed according to the regulations of Vanderbilt University's Institutional Animal Care and Usage Committee. 8 to 12 week mice were used in these studies, including males and females, maintained on normal chow or fed with a Western-type diet containing 21% milk fat and 0.15% cholesterol (Teklad) and drugs (2-HOBA, 4-HOBA) or vehicle. Males and females were studied in separate groups.
Wild animals	No wild animals were used in the study.
Field-collected samples	Study did not involve field-collected samples.
Ethics oversight	Vanderbilt University's Institutional Animal Care and Usage Committee approved the animal protocol in this study.

Note that full information on the approval of the study protocol must also be provided in the manuscript.

Human research participants

Policy information about studies involving human research participants

Population characteristics	The study was approved by the Vanderbilt University Institutional Review Board (IRB), and all participants gave their written informed consent. The human blood from patients with severe familial hypercholesterolemia (FH), who were undergoing LDL apheresis, and healthy controls were obtained using an IRB approved protocol. The study population consisted of both male and female Caucasian subjects with severe FH and healthy control volunteers between 14 to 65 years of age.
Recruitment	The subjects with severe FH were recruited from subjects who were undergoing LDL apheresis at the Vanderbilt University Medical Center after obtaining informed consent using an IRB approved protocol. These subjects have severe FH due to either homozygous FH or severe combined heterozygous FH. There was selection bias in the sense that these subjects have a more severe genetic form of FH than subjects with heterozygous FH. Therefore, the results may differ in subjects with heterozygous FH. However, studies in heterozygous FH subjects have shown that HDL function is impaired in terms of cholesterol efflux capacity. The healthy controls were recruited after obtaining informed consent using an IRB approved protocol. The subjects with severe FH meet the Dutch Lipid Clinic Network diagnostic criteria of definite familial hypercholesterolemia. Both male and female subjects from age 14 to 65 years old were recruited and healthy controls were age and sex matched.
Ethics oversight	The study was approved by the Vanderbilt University Institutional Review Board (IRB)

Note that full information on the approval of the study protocol must also be provided in the manuscript.

Flow Cytometry

Plots

Confirm that:

- [ ] The axis labels state the marker and fluorochrome used (e.g. CD4-FITC).
- [ ] The axis scales are clearly visible. Include numbers along axes only for bottom left plot of group (a 'group' is an analysis of identical markers).
- [ ] All plots are contour plots with outliers or pseudocolor plots.
- [x] A numerical value for number of cells or percentage (with statistics) is provided.

Methodology

Sample preparation	The cell were washed and collected as single suspended cells after treatment
Instrument	The 3-laser BD LSRII (BD Biosciences) configured with 405 nm, 488 nm, and 633 nm lasers was applied.
Software	Fortessa are main analytical platforms and BD Biosciences digital flow cytometry software was applied.
Cell population abundance	2.5 x10^4 cells per batch

Gating strategy	Each time a negative control was always prepared and applied for a validation. Based on their forward and side scatter properties, the Annexin V Alexa Fluor™ 488 positive or negative cells were determined in gating.

☒ Tick this box to confirm that a figure exemplifying the gating strategy is provided in the Supplementary Information.
